# Infrared spectral analysis for the classification of patients with acute coronary syndrome. The questions run so deep

**DOI:** 10.1093/ehjdh/ztad017

**Published:** 2023-03-06

**Authors:** Alessandra Scoccia, Peter de Jaegere

**Affiliations:** Department of Cardiology, Erasmus MC, Dr. Molewaterplein 40, 3015, GD, Rotterdam, The Netherlands; Department of Cardiology, Erasmus MC, Dr. Molewaterplein 40, 3015, GD, Rotterdam, The Netherlands

**This editorial refers to ‘A novel breakthrough in wrist-worn transdermal troponin-I-sensor assessment for acute myocardial infarction’, by S. Sengupta *et al*., https://doi.org/10.1093/ehjdh/ztad015.**

It is said that a journey starts with a first step, a step that often is swept out of our memory or mused to be insignificant. Nonetheless, it may be the kernel of a magnificent experience or discovery. Irrespective of who is at the origin of the idea or who took or made someone take the first step, the paper of Sengupta *et al*. published in this issue of the European Heart Journal—Digital Health is exemplar thereof.^1^

The authors are beyond the critical first step and probably are already halfway. The trip has a determination, namely the classification or differentiation of patients with an acute coronary syndrome (ACS) via the analysis of high-sensitive cardiac troponin-I (Hs-cTnI) level at a single time point using a machine learning (ML) algorithm. Yet, the undisputable innovative part that merits our attention is not the ML technique of analysis but the fact that the authors assessed Hs-cTnI level in the patient’s dermis using a wrist-worn smart sensor assessing the spectral spectrum and, hence, the molecular composition of the interrogated tissue.

Primarily notwithstanding a complex endeavour demanding the input and intelligence of many, the principle of the proposed approach is not so tough to understand. The spectral signature of biologic tissue depends on its chemical composition (e.g. *proteins each with a specific 3D structure and chemical composition and, thus, different physical properties*) and its interaction with incoming energy (e.g. *light at the mid-infrared wavelength*).^2^ Just as a little sidestep, it is reminiscent of the mindboggling relation between matter and energy so astoundingly deduced and elegantly defined by A. Einstein (*m* = *E*/*c*^2^). Back to clinical practice; since the chemical composition of biologic tissue varies along health and disease [e.g. *rise in cardiac markers such as troponin but also myoglobulin and creatine kinase during acute myocardial infarction (AMI)*], mid-infrared spectroscopy allows to differentiate between AMI and non-AMI patients as shown by Petrich *et al*.^3^ who used dried blood in patients with acute chest pain of different causes.

As mentioned above, the authors took a major step forward by introducing a wrist-worn transdermal infrared spectrophotometric sensor (transdermal-ISS), thereby avoiding blood sampling and alluring the potential of a continuous non-invasive monitoring of molecular substances such as Hs-cTnI (*results allegedly available within 5 min*) while capitalizing the potential of the whole chain of digital health (DH), namely automated detection, transfer, stockage, and analysis. What is it that we can dream more of?^4^

The present work must be read while recalling carefully planned earlier work of the authors that consisted of the following: (i) the *ex vivo* evaluation of the spectral features of cardiac biomarkers such as Hs-cTnI in blood samples of 30 patients, (ii) benchtop spectrometer analysis of the spectral pattern of nine patients referred for coronary angiography of whom five with and four without cardiac disease, (iii) the evaluation of the feasibility of the novel point of care (POC) method allowing the finetuning of the infrared detector to the wavelengths corresponding to cardiac troponin-I that was followed by, (iv) a clinical pilot study in which the correlation between smart sensor-derived levels of Hs-cTnI and those standard of care (SOC) via immunoassay was compared in a series of 52 patients with suspicion of ACS.^5^

In the spirit of what DH may offer, the authors took a next logic step, namely the introduction of automated analysis of cardiac biomarkers using a ML algorithm.^1^ In this paper, algorithm performance was assessed by a five-centre pilot study encompassing 238 patients with ACS of whom 20% with unstable angina, 21% non-ST segment elevation acute myocardial infarction (STEMI), and 57% STEMI (*guideline criteria*). The definition of elevated troponin level at a single time point (*yes/no*) was based upon a central lab reference value. The performance of the ML algorithm was assessed using the standard descriptive statistical measures in an internal and external validation cohort of 134 and 45 patients, respectively, elegantly summarized in Table 2.^1^ Overall an AUC-ROC of 90% and higher was observed. In addition, some ancillary analyses have been executed relating the elevated troponin levels with regional wall motion abnormalities on echo and coronary artery stenosis >50% diameter stenosis plus the effect of a number of clinical variables potentially affecting algorithm performance.

It is opening an open door, yet, one needs to be aware and possibly be reminded that the diagnostic performance reported by the authors pertains to the present study given the study population (i.e. *sample size but also patient characteristics that were available upon study entry plus their distribution and/or range in case of continuous variables*) and methods of analysis involving all the steps taken during the entire study process such as—in chronologic order—the classification of the entry criterion (i.e. *ACS*) and its subcategories (i.e. *unstable, non-STEMI, STEMI*), which is the dependent outcome measure of interest. As the authors correctly pointed out, categorization was based upon a laboratory-defined cut-off value of Hs-cTnI taken at a specific point in time of the patient’s disease state while myocardial necrosis is a time-related process. The data taken during all these steps served as input for the ML analysis and, thus, classification.

It is in this background or context that one needs to read the paper and interpret its findings which inescapably leads to the question whether the proposed method is ready for (widespread) clinical implementation or whether more research needs to be done. In addition to the paragraph above, one needs to acknowledge that the outcome of the proposed method not only depends on the study population (i.e. *only those eligible for application of the transdermal-ISS with the exclusion of patients with tremor, skin defects, or wounds*—*moderator effects of biologic covariates presented by the authors—Table 3*) and the ML algorithm but also the device. Similar to every high-tech device, the spectral spectrum generated by the transdermal-ISS is affected by technology-related issues (e.g. *signal-to-noise ratio, the effect of stray light*) and those related to the algorithm(s) used for the data generation and processing.^1,2,4^ These data are then transferred for stockage and analysis. The task of the ML algorithm is to discern in the multitude of data within this spectrum, which is the relation of absorbance (*Y*-axis) with the wavelength or its inverse (wavenumber, *X*-axis), the differences between patients with disease or different diseases or states, which is not a simple task.

With the above, we wish to elucidate the complexity of the work done by the authors for which we express our respect while also offering insights to interpret the findings and so to reach realistic conclusions stimulating next research proposals. The work done by the authors demands an effort, a more in-depth reading, re-reading, and/or digging in other paper as the proposal of the authors involves the whole DH chain.^4^ The Internet of Things, a system of interrelated and connected digital systems and devices that collects, sends, stores, and analyses data over a network without requiring human-to-human or human-to-computer interaction, is a complex field necessitating the brain power of many with different background and origin (e.g. *data and computer scientists, engineers, physicists, physicians*). It is one of the most extraordinary revolutions of this century. It has the undisputed benefit of improving the human condition, through the detection, elaboration, and storage of a constantly increasing volume of multidimensional data that is hard to handle by the human brain.

However, in such subtle things as life and health, it is crucial to remember that machines, even if they will be increasingly important in decision support, will always lack the most important feature: a critical human thinking. The capacity of handling large volumes of multidimensional data using a DH chain (*collection, processing, transmission, analysis*) is astounding and goes beyond our imagination. Yet, one should not underestimate the capacity of the human brain and to give a meaning to findings, in other words, the capacity to interpret. The latter demands an even higher level of intelligence that goes beyond the professed one of artificial intelligence. Furthermore, the beauty of the human brain is that it is capable of being curious and astonished, a set of aptitudes that we may call emotions but so nicely defined by Plato as wonder or ‘the only beginning of philosophy’ as he wrote.^6^

It goes beyond the scope of this editorial to outline philosophy but one can postulate that philosophy transcends natural sciences and that the former starts when the latter reaches its limits in the attempt to understand the natural world (e.g. *life and death, health and disease, and natural phenomena*). As such, they are interrelated or complementary since a spirit capable of transcending the rigour of the scientific matrix of thinking may come with pioneering proposals to solve intricate problems. The convoluted theory of special relativity so nicely captured in the equation above (*m* = *E*/*c*^2^), that is so often proven to be correct, may serve as the most striking example.

The work done by Sengupta *et al*. makes us wonder, wondering where it will lead us, what needs to be done next and wondering in admiration as the proposal is innovative and patient-friendly, tackling a highly relevant clinical problem associated with considerable suffering. It merits our attention and support to bring this proposal to fruition. *Per ardua ad astra*, since the questions still run so deep.
